# The State of Remote Patient Monitoring for Chronic Disease Management in the United States

**DOI:** 10.2196/70422

**Published:** 2025-06-26

**Authors:** Margaret M Paul, Nandita Khera, Praneetha R Elugunti, Kevin C Ruff, Musab S Hommos, Leslie F Thomas, Vivek Nagaraja, Ashley L Garrett, Mari Pantoja-Smith, Nathan L Delafield, Blanca C Lizaola-Mayo, Molly M Kresin, Mahesh Seetharam, Sandhya R Nagarakanti, Manreet Kaur

**Affiliations:** 1 Mayo Clinic in Arizona Phoenix, AZ United States

**Keywords:** remote patient monitoring, chronic disease management, chronic disease, telemedicine, health information systems, reimbursement mechanisms

## Abstract

Remote patient monitoring (RPM) increased exponentially during the COVID-19 pandemic. RPM programs commonly incorporate tools to capture and transmit health-relevant data from the home to the clinical space to augment the clinical decision-making process of health care providers. Given the potential to improve patient health outcomes, health care systems around the world are actively engaged in fashioning, implementing, and exploring the outcomes of various RPM program models. However, new challenges to health care systems include increasing RPM program enrollment, optimizing condition-specific RPM programs to best address the needs of specific patient groups, integrating new RPM-derived data streams into existing IT infrastructure, overcoming limited availability of desired remote monitoring technologies, and quantifying the health outcomes produced by RPM use. Herein, we identify stakeholders for RPM in the United States, summarize the landscape of RPM tools available for chronic disease management, discuss the current regulatory environment, delve into the benefits and challenges of integrating these tools into clinical practice, summarize aspects of coverage and reimbursement, and examine the knowledge and policy gaps regarding sustained use of RPM in clinical practice, along with associated opportunities.

## Introduction

The COVID-19 pandemic catalyzed a significant transformation in health care, marked by substantial advancements in telemedicine [1]. A recent study in the United States showed that Medicare patients in health systems with a relatively high proportion of telemedicine visits had more elective outpatient visits, less-frequent emergency department (ED) visits, and increased adherence to medications prescribed for certain chronic diseases, including diabetes mellitus and hyperlipidemia, as evidenced by the prescriptions filled for metformin and statins [2]. Beyond its potential to improve measures of use, clinical outcomes, cost, and operational efficiency, telemedicine produces higher satisfaction among both patients and health care providers [3]. As the population of individuals with chronic medical conditions increases in the United States, innovation is needed to meet the demand for health care while improving population health. Moving beyond the traditional model of in-person clinical encounters will empower health systems to expand access to care and create new opportunities to improve health outcomes.

This paper examines remote patient monitoring (RPM) for chronic disease management in the United States, where its sustainability depends on uncertain health care payer support, despite a growing evidence base to support its benefits and appeal to users. RPM is a pivotal tool that helps bridge the home-office divide created by routine use of telemedicine. It involves the collection of individual health data outside of the traditional clinical environment, the transmission of such individual health data to health care teams, and the analysis and interpretation of updated data within the context of new or existing care plans to facilitate more optimal disease management [4]. Although RPM existed prior to the COVID-19 pandemic, its incorporation into routine clinical care has expanded significantly since that time, enhanced by supportive legislation [5]. This expanded use has led to a growing knowledge base and new challenges associated with RPM integration and the downstream increase of asynchronous care into traditional clinical operations [1,6]. Still, RPM use continues to increase with innovative approaches to care emerging from the foundation of established tools and existing care delivery pathways. In the context of the US health care system, we identify relevant stakeholders, provide an overview of RPM tools available for chronic disease management across varying levels of patient acuity, discuss the current regulatory environment, delve into the benefits and challenges of integrating these tools into clinical practice, summarize aspects of coverage and reimbursement, and examine the knowledge gaps regarding sustained use of RPM in clinical practice, along with associated opportunities.

## Stakeholders in Digital Technology

A diverse array of stakeholders in the health care ecosystem are impacted by RPM programs. For patients, RPM offers increased convenience in monitoring of certain health metrics outside of the clinical space, allows for increased personal engagement in a portion of the health-monitoring process, and improves both communication and satisfaction related to their care [7]. The financial implications of RPM differ from in-person care, encompassing costs related to medical devices and asynchronous engagement with the care team. Health systems and payers are keenly interested in the degree to which RPM programs impact quality of care and outcomes, especially in the context of value-based care. These groups are also focused on monitoring health care use and cost changes resulting from RPM integration. In our experience, uncertainty in long-term RPM reimbursement creates financial unpredictability for these programs, posing barriers to patient and health system engagement.

Policy makers who regulate RPM at state and federal levels must consider sustainable reimbursement models beyond the pandemic response and address population health implications, including challenges to providing fair access to RPM services to those who may be disadvantaged for varied reasons, including low health literacy, low socioeconomic status, or limited access to technology. Additionally, policy makers must craft reimbursement models that appropriately account for the crucially important “behind the scenes” efforts of the health care team to safely and effectively analyze, interpret, and react to real-time data streams continuously generated by participating patients. Reimbursement models should also be designed in a forward-thinking manner with the explicit realization that the number of chronic health conditions of patients in the US population is increasing while also becoming increasingly complex to effectively manage. Thus, with increasing complexity of the “average” patient within the United States using RPM over time, the safe and effective use of RPM may become more challenging to achieve. Like health systems, medical device companies’ investment in advancing RPM technology depends on effective and long-term reimbursement policies.

## Digital Tools for RPM

RPM tools encompass a variety of technologies designed to remotely monitor patients’ health metrics in real time. Such tools enable patients to actively participate in their health management, generating personal health records and receiving just-in-time feedback from care teams that can enhance quality of care and adherence to treatment plans [8]. RPM tools can be categorized based on the relative frequency of data collection. Devices that collect data relatively infrequently or intermittently include blood pressure measurement devices, pulse oximeters, and handheld point of care glucometers. Devices that collect data at a higher frequency or operate continuously include wearable physical activity trackers, fall-detection sensors, electrocardiography monitors, and continuous glucose measurement devices. Many devices feature wireless data transmission. Table 1 summarizes popular devices by the clinical measures collected and the chronic conditions they are used to monitor. The US Food and Drug Administration (FDA) regulates these devices, many of which received emergency use authorization (EUA) during the pandemic, with a transition plan to normal operations now in place.

**Table 1:** Commonly used digital tools to support remote patient monitoring.

Parameter	Devices	Treatment target
Blood pressure monitoring	Blood pressure monitors	Chronic kidney disease Heart failure High-risk pregnancy
Blood glucose monitoring	Glucometers	Diabetes mellitus
Arrhythmia detection	Wireless electrocardiogram monitoring systems	Atrial fibrillation Cardiac arrhythmia Coronary artery disease
Seizure monitoring systems	Wearable electroencephalogram leads Non-electroencephalogram physiological signal–based seizure monitoring systems	Seizure disorders
Sleep monitoring devices	Continuous positive airway pressure tracking systems	Obstructive sleep apnea
Airway	Peak flow meters Pulse oximeters	Chronic obstructive pulmonary disease Asthma COVID-19 pneumonia
Body weight	Remote monitoring digital weighing scales	Heart failure Decompensated cirrhosis
Body temperature	Remote monitoring digital thermometers	Broad applicability
Fall detection and prevention	Wearable activity and acceleration sensors Ambient thermal sensors	Parkinson disease Postoperative rehabilitation Post-stroke rehabilitation
Postoperative rehabilitation	Smart phone app–based physical therapy rehabilitation programs	Post–orthopedic surgery care

## Considerations for Implementation

The literature on RPM implementation highlights common challenges. RPM correlates with increased in-patient messaging, necessitating updated workflows for electronic “in-basket” management and enhanced asynchronous nonvisit care [6]. Emerging artificial intelligence technologies, such as chatbots to respond to patient messages, promise to reduce staff burden related to these barriers. Patients may face technological barriers to device access and use [9], with broadband limitations further excluding some people [10,11]. RPM implementation and sustainment costs are significant and vary by care plan and device, and cost-sharing with patients poses an additional risk of inequitable program access among economically disadvantaged populations [10]. Implementation strategies to mitigate these common challenges are emerging. For example, a study on an RPM program for maternal hypertension in rural Mississippi addressed technological barriers by providing a comprehensive equipment kit and a one-on-one nurse-led remote education session [12]. Another study based at an urban community health center serving low-income patients found that having a strong physician champion facilitated many aspects of implementation and that developing printed, culturally appropriate educational materials in multiple languages and literacy levels tailored to their population was effective in overcoming initial access barriers for patients [13]. At the health system level, new models of care, such as the RPM usability impact model [14], should be considered to guide implementation activities among patients, as well as caregivers, who play a key role in ensuring that accurate data is captured but are often excluded from targeted educational activities. Best practices have been developed to integrate data acquired via RPM devices into the electronic medical record system [15], though further research is needed to enhance data security [16] and prevent breaches [17].

## Coverage and Reimbursement

In the United States, RPM services are covered by Medicare as communication technology–based services. As of September 2023, 37 state Medicaid programs also provide reimbursement for RPM. The Centers for Medicare and Medicaid Services (CMS), the federal agency that provides health coverage in the United States, has specific billing requirements for RPM. These include an established physician-patient relationship, physiological data monitoring for at least 16 days a month, and use of an FDA-approved device with secure and automated data upload. RPM reimbursement codes cover fees for initial enrollment (Current Procedural Terminology [CPT] code 99453), monthly data monitoring (CPT code 99454) and health care provider time for communicating with patients (CPT codes 99457 and 99458) [18].

Alongside the CMS, a growing number of commercial payers reimburse for RPM [19], albeit with disparities across plans. Inconsistencies in RPM reimbursement produce differential access to these programs, which are most readily accessible by patients capable of participating without insurance support (eg, to access devices and training without additional or significant costs). In addition, as these programs see wider implementation, reliable monitoring of the effort required by health care teams to manage them is needed and may in turn influence reimbursement mechanisms and out-of-pocket costs for patients. Reimbursement models that establish parity across modes of care are needed to incentivize RPM use, rather than penalize health care providers and patients for lack of the traditional in-person office visit.

Traditionally, RPM services are provided in conjunction with chronic care management for low-acuity care, with the goal of preventing disease exacerbation or hospitalization. Patients with chronic illnesses, such as congestive heart failure, advanced kidney disease, advanced liver disease, hypertension, and diabetes mellitus, are naturally suited for RPM programs. Similarly, patients recovering from an acute event (stroke, major surgery, or hospitalization for an exacerbation of a chronic illness) benefit from RPM programs that aim at preventing hospital readmission and that allow safe discharge planning. In addition to disease-specific groups, patients who are geographically isolated or have limited transportation and social support may benefit from the improved access to care RPM provides.

## Regulatory Implications for Quality And Safety

The increasing uptake of RPM tools has prompted the creation of Health Insurance Portability and Accountability Act–compliant digital platforms capable of integrating data from multiple RPM data streams to generate a comprehensive patient profile. RPM devices and associated software are regulated by the FDA as medical devices, and in October 2023, the FDA issued updated guidelines for their approval and regulation [20]. The update reflects a transition from the EUA issued in March 2020 during the COVID-19 public health emergency, thereby reinforcing the FDA’s commitment to maintaining enforcement policies for RPM tools beyond the pandemic.

FDA regulation applies to all devices that can measure physiological parameters accurately and noninvasively and that can be connected to a wireless network to transmit patient data securely to the health care provider. Medical devices in the United States are regulated under applicable parts of Title 21 of the US Code of Federal Regulations, which categorizes devices into one of three classifications (class I, class II, and class III), where the classification level depends on the specifics of the function of the device and its intended use and operators to assure the safety and efficacy of the device. Manufacturers of regulated devices are required to maintain a compliant quality management system covering design and development and premarket and postmarket activities related to the device and are subject to annual audits of regulatory compliance. Class I nonexempt and class II devices are cleared by the FDA after review of a de novo submission supporting safety and efficacy of the device or after review of a 510(k) submission supporting substantial equivalence to an existing, legally marketed predicate device. As a point of reference, point-of-care capillary blood glucose measurement devices (ie, glucometers) are invasive, class II medical devices. Class III devices are approved by the FDA after a premarket approval submission demonstrating safety and efficacy of the device for its stated intended use and indications for use. In 2023, the FDA released guidance indicating that it does not enforce submission of additional regulatory applications for devices already in use that undergo minor modifications to their indication for use and functionality to enable them to be used for RPM [20].

## Knowledge Gaps and Opportunities

RPM has the potential to fundamentally transform chronic disease management and patient–health care provider interactions. While its expanded use began as a pandemic response, its sustained integration into clinical practice suggests long-term viability [2]. Emerging evaluations of RPM programs indicate increasing acceptance among patients and health care providers, improved adherence to care plans by patients, and favorable impacts on health care quality and clinical outcomes in line with in-person care [1,13,21]. However, iterative improvements in RPM program designs are needed to enable more seamless application in heterogeneous patient populations, better cost-effectiveness, and improvements in quality of care and outcomes and patient and health care provider satisfaction. Additional research is needed to understand implementation strategies that account for access issues that, if left unresolved, could lead to inequitable access to these programs.

Health care is following in the footsteps of many other industries whose services reach consumers in their own homes. As we strive for increasing uptake and sustainability of these programs at the Mayo Clinic, we are expanding our virtual care offerings to encompass additional disease groups. Two challenges to accomplishing this in our own system are (1) a lack of interoperability and, relatedly, a significant administrative burden for our clinicians due to the need for manual information input and (2) the need to log in to two different systems, which could potentially lead to mistakes. The implementation of these initiatives has presented other considerable challenges, and we are actively identifying barriers to patient enrollment and exploring potential solutions in collaboration with our patients and health care providers. However, continued financial viability and equitable patient access will be determined by longer-term policy decisions. Further research is needed to explore data integration methods that address security concerns, identify implementation strategies to mitigate potential inequities in access, and examine how advancements in artificial intelligence can alleviate care team burdens associated with increased patient communication. Permanent reimbursement models like those established for behavioral and mental health services, which account for equity and quality of care, could ensure that RPM remains an accessible and sustainable option for all patient populations, including those in resource-constrained settings [22,23]. RPM has the potential to be transformative for chronic and acute care as long as barriers and challenges facing RPM implementations can be addressed.

## Abbreviations

CMS: Centers for Medicare and Medicaid Services
CPT: Current Procedural Terminology
ED: emergency department
EUA: emergency use authorization
FDA: Food and Drug Administration
RPM: remote patient monitoring
